# Toward Power Analysis for Partial Least Squares‐Based Methods

**DOI:** 10.1002/bimj.70050

**Published:** 2025-03-13

**Authors:** Angela Andreella, Livio Finos, Bruno Scarpa, Matteo Stocchero

**Affiliations:** ^1^ Department of Economics and Management University of Trento Trento Italy; ^2^ Department of Statistical Sciences University of Padova Padova Italy; ^3^ Department of Women's and Children's Health University of Padova Padova Italy

**Keywords:** classification, omics data, partial least squares, permutation tests, power analysis

## Abstract

In recent years, power analysis has become widely used in applied sciences, with the increasing importance of the replicability issue. When distribution‐free methods, such as partial least squares (PLS)‐based approaches, are considered, formulating power analysis is challenging. In this study, we introduce the methodological framework of a new procedure for performing power analysis when PLS‐based methods are used. Data are simulated by the Monte Carlo method, assuming the null hypothesis of no effect is false and exploiting the latent structure estimated by PLS in the pilot data. In this way, the complex correlation data structure is explicitly considered in power analysis and sample size estimation. The paper offers insights into selecting test statistics for the power analysis procedure, comparing accuracy‐based tests and those based on continuous parameters estimated by PLS. Simulated and real data sets are investigated to show how the method works in practice.

## 1 Introduction

Nowadays, scientists are increasingly forced by ethical and economic considerations to apply power analysis for sample size estimation since inferential statistics can only lead to robust and reliable results by implementing the right experimental design. Indeed, the number of observations should not be too large for efficiency, ethical, and cost reasons but enough to guarantee reliable statistical results with minimal false positive or false negative rates. Several authors (e.g., Button et al. 2013; Ioannidis 2005) highlighted that the results of many published biomedical studies are unreliable and probably false due to the small sample size used, and most of the published omics studies are underpowered.

In particular, when analyzing multivariate data, the responses are typically correlated, redundant, and noisy, and the number of observations is generally smaller than the number of responses. In this framework, likelihood‐free approaches such as partial least squares (PLS)‐based methods (Wold et al. 2001, 1983) are applied, and traditional techniques for power analysis cannot be used for sample size estimation. In addition, methods such as PLS, as well as canonical correlation analysis (CCA, Hotelling 1992; Jordan 1875) and principal component analysis (PCA, Jolliffe 2002; Pearson 1901), are generally viewed as exploratory methods rather than as testing procedures (Winkler et al. 2020). This means that the model parameters are uncommonly interpreted as statistical effects, and no single definition for the effect size is available. For these reasons, we propose here a methodology for performing power analysis when PLS‐based methods are used as tools for data analysis.

Since PLS‐based methods are not based on statistical distributions and are not likelihood estimation‐based techniques, strategies for power analysis and sample size estimation should be then based on numerical simulation. One method common in the literature is using Monte Carlo (MC) simulation (Muthén and Muthén 2002; Martens et al. 2000) that generates a large set of artificial data using the design under evaluation to design hypothetical experiments. The artificial data are analyzed in the same way as the real data set, obtaining the distributions of the model parameters of interest. Then, the optimal sample size is estimated by studying the cost and risks of Type I and Type II errors associated with the given experimental design.

To the best of our knowledge, only a few studies investigated the problem of power and sample size estimation in the case of PLS‐based techniques. Blaise et al. (2016) introduced an interesting approach based on MC simulation where the correlation between variables is explicitly incorporated. Specifically, new samples with marginal distributions and correlation structures similar to the ones observed in the pilot data are simulated, modeling the log‐transformed pilot data as a multivariate normal distribution. Thus, the effect size is introduced by acting on subsets of correlated variables. The relationship between statistical power, sample size, and effect size is investigated by analyzing the artificial data and obtaining the distributions of the statistics of interest. In a second study, Saccenti and Timmerman (2016) drew a possible line of thought to perform power analysis for PCA and PLS for discriminant analysis (PLS‐DA). Important concepts have been discussed, and some interesting ideas have been offered for sample size estimation in a multivariate setting. Specifically, in the case of PCA, they proved that inference and sample size estimation could be grounded by solid statistical characterization of the distributional properties of the PCA solution, while for PLS‐DA, the scenario is more complex. In another study, Nyamundanda et al. (2013) proposed a general method for sample size estimation based on simulated data generated from probabilistic principal component analysis (PPCA)‐based models that can be applied without experimental pilot data. The approach considers only univariate data analysis controlling false discovery rate as a data analysis strategy and does not examine PLS‐based methods.

In this manuscript, we present a novel method for power analysis that uses the score structure discovered by PLS from the pilot data to simulate new data sets with the same covariance structure of the pilot data but different sample sizes. This is one of the main novelties of our study. Indeed, unlike the above‐mentioned published approaches, data simulation used in power analysis is performed here to see the data from a multivariate point of view, respecting the correlation structure. The same strategy PLS uses to model the data is then applied in the data simulation process. This makes the approach tailored explicitly for PLS. Another novelty concerning the above‐mentioned published methods is that different test statistics for the power analysis procedure are proposed and investigated. Moreover, the approach is developed as R package to ensure accessibility for researchers, enhancing transparency and replicability of the results.

For the sake of simplicity, we consider the simple case of a case–control study that is investigated employing PLS for classification (PLSc). The reason for focusing our attention on this simple case is twofold. First, the case–control setting is one of the most largely implemented study designs in omics sciences and beyond. Second, PLSc in the limit case of a 2‐class problem is a simple but not trivial example of a PLS‐based technique that can be used both to show how our new approach can be formulated and to discuss the use of different test statistics. So, three test statistics are considered to analyze the null hypothesis of equal distribution between the two classes. The first test statistic is based on the Matthews correlation coefficient (MCC), widely used in the PLS analysis framework. Since the hypothesis testing based on this test statistic loses power due to its discretization nature (Rosenblatt et al. 2021), we propose a two‐sample t‐test based on the predictive score matrix estimated by PLS and the squared Pearson correlation coefficient properly transforming the categorical dependent variable.

The proposed method can be extended to other PLS‐based techniques following the same lines of thought.

The paper is structured as follows. Section 2 summarizes the theory underlying PLS to provide a background to understand the following sections. Section 3 defines the permutation‐based test statistics to analyze the null hypothesis of equal distribution between the two classes. Section 4 shows the strategy for power analysis and sample size estimation based on MC simulation. Section 5 briefly discusses the use of the test statistics calculated by cross‐validation. Section 6 analyzes simulated and real data sets to show how the method works in practice. Discussion and concluding remarks are reported in Section 7.

Without loss of generality, we consider that the data are mean‐centered throughout the manuscript unless stated otherwise. In addition, the notation ${∥A∥}_{F}$ stands for the Frobenius norm of a general matrix A.

## 2 Background

The theoretical framework of PLS for regression (PLSR, Wold et al. 1983) and PLSc (Stocchero et al. 2021) is briefly presented to give the reader the helpful background to understand the procedure for power analysis and sample size estimation introduced in Sections 3 and 4.

### 2.1 PLS for Regression

Let $Y\in R^{N\times K}$ be the matrix of dependent variables and $X\in R^{N\times P}$ the matrix of predictor variables, where N is the number of observations, K the number of dependent variables, and P the number of independent ones. Considering the linear regression model Y=XB+F where F is the error term matrix with mean 0 and covariance matrix Σ, PLSR estimates the coefficient matrix B decomposing the X and Y matrices by means of the scores matrix T, as defined in the following definition.

Definition 2.1 Let be $Y\in R^{N\times K}$ and $X\in R^{N\times P}$. PLSR can be rephrased in terms of the score matrix T as:

$$\begin{array}{cc} X & =TP^{⊤}+E,\\ Y & =TQ^{⊤}+F, \end{array}$$

where E, F are error terms matrices, and $P=X^{⊤}T{\left(T^{⊤}T\right)}^{-1}$ and $Q=Y^{⊤}T{\left(T^{⊤}T\right)}^{-1}$ the loadings matrices of X and Y, respectively.

An iterative procedure, the so‐called PLS2 algorithm calculates the scores matrix. At each iteration a∈{1,⋯,A} with A∈{1,⋯,rank(X)} of the algorithm, a suitable weight vector $w_{a}$ is calculated as solution of the following eigenvalue problem ${\hat{E}}_{a-1}^{⊤}{\hat{F}}_{a-1}{\hat{F}}_{a-1}^{⊤}{\hat{E}}_{a-1}w_{a}=\lambda_{a}w_{a}$ where ${\hat{E}}_{a-1}$ and ${\hat{F}}_{a-1}$ are the residual matrices calculated in the previous iteration a−1 and $\lambda_{a}$ is the eigenvalue associated with $w_{a}$. The weight vector is then used to project the residual matrix of X to obtain $T=\left[t_{a}\right]=\left[{\hat{E}}_{a-1}w_{a}\right]\in R^{N\times A}$. We have used [·] to stand the generic column vector of the referred matrix.

At the first iteration, ${\hat{E}}_{0}:=X$ and ${\hat{F}}_{0}:=Y$ whereas, after A iterations, the final residual matrices are $\hat{E}:={\hat{E}}_{A}$ and $\hat{F}:={\hat{F}}_{A}$. The complete PLS2 algorithm is reported in the pseudocode defined in Algorithm A1.

It is worth noting that T is a linear combination of the columns of X and that the complexity of the model depends on the number A of iterations, which is then extremely important in PLS. Indeed, given the number A of iterations, the PLS model is completely defined. Moreover, the number A is also the rank of the score matrix T and of the weight matrix W, having both orthogonal columns (Stocchero 2019; Höskuldsson 1988).

The matrix of the regression coefficients B is then estimated as $\hat{B}=W{\left(W^{⊤}X^{⊤}XW\right)}^{-1}W^{⊤}X^{⊤}Y$ and $W=\left[w_{a}\right]\in R^{P\times A}$ after A iterations. In the case of a full column rank matrix of the predictor variables, it can be shown that $\hat{B}$ is a biased estimator of B since $E\left(\hat{B}\right)=W{\left(W^{⊤}X^{⊤}XW\right)}^{-1}W^{⊤}X^{⊤}X{\hat{B}}_{\text{OLS}}$ where ${\hat{B}}_{\text{OLS}}$ is the ordinary least squares (OLS) estimator. In addition, $\hat{B}={\hat{B}}_{\text{OLS}}$ if the number of score components equals the number of predictor variables, that is, A=P. In the general case of a rank‐deficient matrix of the predictor variables, when the maximum number of iterations is performed, the matrix of the regression coefficients is $\hat{B}=V_{X}S_{X}^{-1}U_{X}^{⊤}Y$, where the singular value decomposition (SVD) $X=U_{X}S_{X}V_{X}$ has been considered and solves the least squares problem. Pulling $\hat{B}$ away from the least squares solution helps balance the trade‐off bias–variance, leading to approximations that better predict new observations.

The columns of T can be used as coordinates to represent the observations in a space with dimension A. Since A≪P in most cases, PLSR produces an efficient data reduction that simplifies the investigation of the data variation of X explaining Y. Unfortunately, A is often greater than rank(Y), and then, the matrix factorization of Y of Definition 2.1 becomes suboptimal, that is, a higher number of dimensions is being used to represent the data than is actually necessary. To overcome this suboptimality, Stocchero and Paris (2016) proposed an alternative matrix factorization by applying a post‐transformation procedure.

Post‐transformation is a procedure that, starting from a PLS model, generates a new PLS model where the score space is partitioned into two orthogonal subspaces. The first one is the predictive subspace spanned by the predictive score matrix called $T_{P}$ correlated to the dependent variables. The second one is the nonpredictive subspace described by the nonpredictive score matrix called $T_{O}$ orthogonal to Y. So, the number of predictive score vectors equals rank(Y), and the data variation explaining the dependent variables is included exclusively in the predictive part of the model. Post‐transforming the PLSR model returns the following matrix factorization:

$$\begin{array}{cc} X & =T_{P}P_{P}^{⊤}+P_{O}T_{O}^{⊤}+E,\\ Y & =T_{P}Q_{P}^{⊤}+F, \end{array}$$

where $P_{P}$ and $P_{O}$ are, respectively, the predictive and orthogonal loadings matrices of X, and $Q_{P}$ the predictive loading matrix of Y. Interestingly, $\hat{E}$ and $\hat{F}$, and $\hat{B}$ are the same of the original PLSR model. The set of predictive scores can then be used as bases to build the latent variables explaining the dependent variables. More details about the post‐transformation procedure are in Appendix B.

Most of the techniques of the PLS family can be generated from the PLS2 algorithm used to perform PLSR by modifying the equation for the weight calculation (e.g., including constraints the orthogonally constrained PLS version is obtained (Stocchero et al. 2018)), or introducing suitable dependent variables (e.g., coding the categorical variable with dummy variables, the PLS‐DA (Barker and Rayens 2003) is obtained). Moreover, all those techniques that are based on the iterative deflation algorithm (IDA) (see Appendix A) can be post‐transformed (Stocchero 2019).

### 2.2 PLS for Classification

PLSc (Stocchero et al. 2021) is an adaptation of PLSR when the support of Y equals Y={1,⋯,G} (i.e., a G‐class problem with G number of classes). For the sake of simplicity, we consider here the case of a 2‐class problem. However, the approach can be easily extended to the case of G>2.

Given $N_{1}$ observations of class 1 and $N_{2}$ observations of class 2 such that $N=N_{1}+N_{2}$, we define the blockwise probability‐data matrix having dimension N×2 as:

$$Z=\left[\begin{array}{cc} \left(1-\varepsilon \right)1_{N_{1}} & \varepsilon 1_{N_{1}}\\ \varepsilon 1_{N_{2}} & \left(1-\varepsilon \right)1_{N_{2}} \end{array}\right],$$

where $1_{d}$ is a vector of d ones, and ε<1/2. Mean centering Z by its columnwise mean $\bar{Z}$ and applying the isometric log‐ratio transformation ilr(·), the vector

(1) $$f_{0}=\text{ilr}\left(Z⊖\bar{Z}\right)$$

is obtained. The symbol ⊖ indicates the subtraction in the simplex $S^{2}=\{[Z_{n}^{⊤}]\in R^{2}:Z_{\mathrm{ng}}>0,\sum_{g=1}^{2}Z_{\mathrm{ng}}=1\}$, where $Z_{ng}$ is the generic elements in row n and column g of the probability‐data matrix Z. In other words, $f_{0}=\left(Z⊖\bar{Z}\right)H^{⊤}$ where H is a G−1×G orthonormal matrix and the rows are orthogonal to $1_{G}$ vector of ones (Tsagris et al. 2011). The PLSc can be formulated as the regression problem $f_{0}=XB+F$.

The matrix of the estimated regression coefficients $\hat{B}$ is obtained using the PLS2 algorithm explained in Subsection 2.1 considering $f_{0}$ defined in Equation (1) instead of Y. The two‐dimensional probability‐data vector for a given observation $x_{n}\in R^{P\times 1}$ with n∈{1,⋯,N} is calculated by $\left[{\hat{Z}}_{n}^{⊤}\right]={\text{ilr}}^{-1}\left(x_{n}^{⊤}\hat{B}\right)⊕\left[{\bar{Z}}_{n}^{⊤}\right]$ where ⊕ stands for the addition in the simplex $S^{2}$. Finally, the estimated class membership ${\hat{g}}_{n}$ for a given observation n∈{1,⋯,N} is the arguments of the maxima of $\left[{\hat{Z}}_{n}^{⊤}\right]$, that is, ${\hat{g}}_{n}={\text{argmax}}_{g}\lim_{\varepsilon \to 0^{+}}\left[{\hat{Z}}_{n}^{⊤}\right]$ which is independent of the value of ε (Stocchero et al. 2021).

The model can be post‐transformed by applying the same procedure presented for the PLS2 algorithm using $f_{0}$ instead of Y. Specifically, post‐transformation returns a single vector of predictive scores in the case of a 2‐class problem, independently of the transformation used to map the class into the Euclidean space. As a general result, post‐transforming the PLSc model leads to G−1 predictive score components for a given G‐class problem.

## 3 Statistical Test for PLS‐Based Methods

For the sake of simplicity, we consider here again a 2‐class problem, but the methodology can be extended to more complex problems as specified in Section 7. Let us denote with $X_{g}$ the matrix of the predictor variables regarding the g∈{1,2} class, and with $X_{1}$ and $X_{2}$ the distributions of $X_{1}$ and $X_{2}$, respectively. Within the PLSc framework, the null hypothesis $H_{0}:X_{1}=X_{2}$ is tested to evaluate if the predictor variables are similarly distributed between the two classes. In principle, various test statistics can be used to test $H_{0}:X_{1}=X_{2}$. Here, we propose three different test statistics, which are then utilized in hypothesis tests whose power is analyzed in Section 6.

### 3.1 Accuracy‐Based Test Statistic

The first test statistic presented here is based on the MCC, equivalent to the normalized Pearson $\chi^{2}$ statistic, calculated considering the contingency table obtained with the real class, a common choice in PLS literature. Due to its discretization nature, the hypothesis test using the MCC‐based test statistic suffers from low power, that is, it is less sensitive to mild perturbations of the data (Rosenblatt et al. 2021). We clarify here that we do not consider other measures related to the confusion matrix, such as sensitivity, specificity, precision, and negative predictive values. In our context, the false positives and negatives are equally important in the power calculation; the MCC is then a more coherent and reliable metric for evaluating binary classifications (Chicco and Jurman 2023). However, other metrics may be more suitable for guiding decisions in applied settings such as medical diagnostics, where false positives and negatives carry different implications. Although these considerations fall outside the scope of our study, they highlight the importance of choosing performance metrics that align with the specific goals and constraints of the application domain. So, for example, if the aim is to classify a particular g class correctly, indices like the $F_{1}$ score (Chen et al. 2004) and Fowlkes–Mallows one (Fowlkes and Mallows 1983) can be used instead of the MCC. Although we focus exclusively on the MCC in this work, the associated R package powerPLS implements additional metrics, including sensitivity, specificity, and the $dQ^{2}$ index proposed by Westerhuis et al. (2008).

### 3.2 Two‐Group Test Statistics

The second test statistic proposed here is based on the predictive score vector $T_{P}$. Under $H_{0}:X_{1}=X_{2}$, we have $T_{1P}=T_{2P}$ where $T_{gP}\in R^{N_{g}\times 1}$ is the distribution of the predictive scores considering the class g∈{1,2}. The test statistic is defined as a two‐sample t‐test considering the predictive scores for each class g as samples. The hypothesis test using this test statistic is, in principle, more powerful than the one based on the MCC because it overpasses the discretization problem, as it will be seen in the simulation analysis presented in Section 6.1.

The third one is the squared Pearson correlation coefficient $R^{2}$ between the observed dependent variable $f_{0}=\text{ilr}\left(Z⊖\bar{Z}\right)$ defined in Equation (1) and the estimated one $X\hat{B}$. As per the score‐based test statistic, the hypothesis test using $R^{2}$ is, in principle, more powerful than the one based on MCC, overpassing the discretization problem, even if both $R^{2}$ and MCC are based on the estimated matrix of the regression coefficients. In fact, the $R^{2}$ statistic analyzes the correlation between the estimated and observed probability‐data matrix transformed in real space by the isometric log‐ratio transformation. Instead, the MCC test uses the final class membership directly. Finally, the $R^{2}$ can be utilized as the test statistic in PLS for both regression and multiclass classification contexts.

### 3.3 Permutation Test

Let us denote with T one of the three test statistics proposed above. We rely on its permutation distribution to compute the corresponding p‐values. Let us define with P the set of all possible permutation matrices; we randomly select J permutation matrices $P_{j}\in P$ where 1≤j≤J≤|P|. Since under $H_{0}:X_{1}=X_{2}$ the observations are exchangeable, we can randomly permute J times the class labels to compute the null distribution of T, that is, we consider the transformation $P_{j}Y$ (Commenges 2003). We fix as first transformation $P_{1}$ the identity one to get exact α control (Hemerik and Goeman 2018; Pesarin 2001). This way, the p‐value can never equal 0 since $T_{1}$ is the observed test statistic, and the achievable α equals 1/J. Let us consider the test statistic T computed under transformation j of the data as $T_{j}$ with j∈{1,⋯,J}, the p‐value is simply calculated as

(2) $$p=\frac{\sum_{j=1}^{J}I_{T_{j}\geq T_{1}}}{J}$$

considering a right‐tailed rejection region. If the p‐value is less than the given significance level α, we declare $X_{1}\neq X_{2}$.

## 4 Power Analysis

Since PLS‐based methods are not based on statistical distributions and are not likelihood estimation‐based methods, strategies for power analysis should be based on numerical simulation. We propose here an approach to simulate data under the alternative hypothesis (Subsection 4.1) and the complete procedure to estimate power and sample size (Subsection 4.2), considering the test statistics presented in Section 3.

The power and sample size are estimated based on the given pilot data with a fixed number of scores A, assuming an effect is present.

### 4.1 Simulate Data Under the Alternative Hypothesis

The PLS model of the pilot data is used to simulate new data sets with a given sample size $\tilde{N}$, which are in turn used to calculate the power of the hypothesis test based on the test statistic T defined in Section 3. The effect size is assumed to be the same captured by the PLS model of the pilot data and is not modified during the simulation. Moreover, since PLS techniques exploit the correlation structure underlying X, the covariance structure of the pilot data should be preserved when new data are simulated under the alternative hypothesis.

In the following, we define the proper simulation model.

Definition 4.1 Considering the PLS model of Definition 2.1 and the pilot data X and Y, the matrix $\tilde{X}$ of the simulated data under the alternative hypothesis $H_{1}$ is defined as $\tilde{X}=\tilde{T}P^{⊤}+\tilde{E}$ where $\tilde{T}$ is the score matrix under $H_{1}$, $P=X^{⊤}T{\left(T^{⊤}T\right)}^{-}$ is the loading matrix calculated by the PLS model of X and Y, and $\tilde{E}$ is the simulated residual matrix.

It is worth noting that the number of observations of the simulated data can differ from that of the pilot data. The following theorem defines the constraints that $\tilde{T}$ and $\tilde{E}$ must satisfy in order to preserve the covariance structure of the pilot data X.

Theorem 4.2 Considering the model of Definition 4.1 and that of Definition 2.1, under the assumptions $∥TP^{⊤}{∥}_{F}\gg {∥E∥}_{F}$ and $∥\tilde{T}P^{⊤}{∥}_{F}\gg {∥\tilde{E}∥}_{F}$, if ${\tilde{T}}^{⊤}\tilde{T}=T^{⊤}T$ and ${\tilde{T}}^{⊤}\tilde{E}=0$ then $X^{⊤}X\approx {\tilde{X}}^{⊤}\tilde{X}$.

Let us consider the PLS model of Definition 2.1. Since $PT^{⊤}$ is orthogonal to $\hat{E}$, the covariance matrix of X equals $\text{cov}\left(X\right)\propto X^{⊤}X=PSP^{⊤}+E^{⊤}E$ where $S=T^{⊤}T$. So, $\text{cov}\left(X\right)\approx PSP^{⊤}$ when

(3) $$∥TP^{⊤}{∥}_{F}\gg {∥E∥}_{F},$$

that is, in the presence of negligible noise.

Analogously, considering Definition 4.1, one has

$$\text{cov}\left(\tilde{X}\right)\propto {\tilde{X}}^{⊤}\tilde{X}=P{\tilde{T}}^{⊤}\tilde{T}P^{⊤}+{\tilde{E}}^{⊤}\tilde{E}+{\tilde{E}}^{⊤}\tilde{T}P^{⊤}+P{\tilde{T}}^{⊤}\tilde{E}.$$

If ${\tilde{T}}^{⊤}\tilde{T}=S$ and ${\tilde{T}}^{⊤}\tilde{E}=0$, under the condition $∥\tilde{T}P^{⊤}{∥}_{F}\gg {∥\tilde{E}∥}_{F}$ the simulated data $\tilde{X}$ and the pilot data X show the same covariance structure.□

If some data structures are still present in the residual matrix, condition (3) may not be satisfied. In this case, the residual matrix can be modeled by PCA, and the obtained scores and loadings can be included in the matrix factorization of X generated by PLS. However, suppose the PLS‐based model exhibits a high $R_{X}^{2}$ (i.e., a correlation coefficient measuring the proportion of variance in X explained by the estimated latent scores), condition (3) will likely be satisfied in that case, as the residuals would be negligible.

To guarantee that ${\tilde{T}}^{⊤}\tilde{T}=T^{⊤}T$ and ${\tilde{T}}^{⊤}\tilde{E}=0$, the following procedure is here proposed to simulate a set of $\tilde{N}$ observations. We construct the matrix $\tilde{\tilde{T}}\in R^{\tilde{N}\times A}$ sampling from the multivariate distribution of the PLS scores, which is estimated, for example, using kernel density estimation‐based approaches (Sheather 2004). This distribution includes the scores from the PCA model of the residuals if needed. In general, the scores in $\tilde{\tilde{T}}$ are not orthogonal, and its covariance structure is different from that of T. As a consequence, a suitable orthogonalization procedure must be applied. Considering the SVD $\tilde{\tilde{T}}\left(T^{⊤}T\right)=UDV^{⊤}$, the matrix $\tilde{T}\in R^{\tilde{N}\times A}$ is calculated by $\tilde{T}=UV^{⊤}{\left(T^{⊤}T\right)}^{1/2}$.

Thus, the residual matrix $\tilde{E}$ is calculated as follows. The rows of the residual matrix of the PLS model $\hat{E}$ (or those of the residual matrix after PCA modeling of the PLS‐residual matrix if needed) are sampled with replacement $\tilde{N}$ times to obtain the rows of the new matrix $\tilde{\tilde{E}}$ that is made orthogonal to $\tilde{T}$ by projection as $\tilde{E}=\left(I_{n}-\tilde{T}{\left({\tilde{T}}^{⊤}\tilde{T}\right)}^{-1}{\tilde{T}}^{⊤}\right)\tilde{\tilde{E}}$. Once $∥\tilde{T}P^{⊤}{∥}_{F}\gg {∥\tilde{E}∥}_{F}$ is numerically tested, it can be proved that the proposed procedure leads to scores and residuals that satisfy Theorem 4.2.

The procedure here introduced is general and can be used both for regression and for classification problems.

The estimation of the dependent variables in $\tilde{Y}$ associated with the $\tilde{N}$ observations and predictor variables $\tilde{X}$ depends in general on the support Y. In the case of Y={1,⋯,G} with G>2, the class of the new observations can be assessed based on the distributions in the score space of the pilot data, partitioning that space by class. For instance, in the simple case of Y={1,2}, new observations for a given class are simulated by sampling the score distribution of the observations of that class for the pilot data. In the case of Y=R, the dependent variable may be estimated using the PLS model of the pilot data to predict the new simulated observations, adding an error term calculated sampling the distribution of the error term of the pilot data, but this case is out of the aim of the present study.

Finally, while generating data with the same mean and correlation structure as the pilot data might seem simpler, our approach addresses the challenge of identifying the latent structure of the pilot data and generating new data based on this structure using PLSc. Thus, we provide a distribution‐free, data‐dependent method that offers greater flexibility and accuracy.

### 4.2 Power and Sample Size Calculation

Given the procedure that allows the simulation of new data under the alternative hypothesis (Section 4.1), the test statistic T introduced in Section 3, and assuming a significance level α, a number of score components A and a sample size $\tilde{N}$, the power is estimated applying the pseudocode defined in Algorithm 1.

ALGORITHM 1 **The pseudocode shows the procedure to estimate the power associated with a PLS model with A score components considering a data set with $\tilde{N}$ observations given the significance level α, the number of simulations I used in MC simulation and the number of permutations J used to estimate the p‐value for the hypothesis test based on the test statistic T defined in Section 3.**

**Table:** 

**Require:** X, Y, A, $\tilde{N}$, T, α, I, J ▹ X, Y are the pilot data.	
**Ensure:** power	
1:	power←0
2:	**for** i in 1,⋯,I **do**
3:	$\tilde{X}$, $\tilde{Y}$ ← simulate $\left(X,Y,\tilde{N}\right)$ ▹ simulate $\tilde{N}$ samples under $H_{1}$ following the procedure defined in Section 4.1 using the pilot data X, Y and A latent score components
4:	Compute $\mathrm{PLS}(\tilde{X},\tilde{Y},A)$ ▹ compute the PLS model using $\tilde{X}$, $\tilde{Y}$ and A components
5:	Compute $T_{1},\text{…},T_{J}$ ▹ compute the null distribution of T using the PLS results from step 4
6:	$p=\frac{\sum_{j=1}^{J}I_{T_{j}\geq T_{1}}}{J}$ ▹ compute p‐value
7:	**if** Ap≤α **then**
8:	power←power+1/I ▹ compute power
9:	**end if**
10:	**end for**

The procedure is general and can be applied both to classification and to regression problems once a suitable statistical test procedure is introduced.

It is worth noting that considering a PLS model with more than one score component, the permutation‐based p‐values (described in Equation (2) and calculated in row 6 of Algorithm 1) must be corrected for multiplicity to control the familywise error rate (FWER, Goeman and Solari 2014). Indeed, considering A score components and the Bonferroni method, the adjusted p‐value is ${\tilde{p}}_{A}=Ap_{A}$ where $p_{A}$ is the p‐value related to $H_{0}:X_{1}=X_{2}$ when A score components are considered.

Given the procedure for power calculation defined in Algorithm 1, the sample size estimation can be performed following the procedure described in the pseudocode of Algorithm 2.

ALGORITHM 2 **The pseudocode shows the procedure to estimate the optimal sample size $\hat{N}$ for a PLS model with A score components given the significance level α and power level 1−β; I is the number of simulations used in power calculation and J the number of permutations used to estimate the p‐value for the hypothesis test based on the test statistic T defined in Section 3. The algorithm takes as initial candidate value $N_{\min}$.**

**Table:** 

**Require:** X, Y, A, $N_{\min}$, T, α, β, I, J ▹ X, Y are the pilot data.	
**Ensure:** $\hat{N}$	
1:	$n\leftarrow N_{\min}$
2:	power(n)←calculate power(X,Y, A,n,T,α,I,J) ▹ use Algorithm 1
3:	**while** power(n)≥1−β **do**
4:	n←n+1
5:	power(n)←calculate power(X,Y, A,n,T,α,I,J) ▹ use Algorithm 1
6:	**end while**
7:	$\hat{N}\leftarrow n$

Algorithm 1 involves several computationally intensive steps. One of the primary time‐consuming components of Algorithm 1 is Step 3, which estimates the distribution of the PLS‐score matrix. To address this, we employed a fast implementation of the kernel density estimation process as described by Hofmeyr (2019), reducing the time complexity of Step 3 (repeated I times) in Algorithm 1 from O(INP) to O(I(N+P)), where I is the number of simulations used in the power calculation. In addition, the for loop of Algorithm 1 can be parallelized across i∈{1,⋯,I} to enhance efficiency.

Another time‐consuming function in Algorithm 1 is the eigenvalue PLS2 algorithm, detailed in Algorithm A1 in Appendix A. This algorithm has a time complexity of $O(AP^{2})$. Given that typically P≫N, the overall time complexity of the approach is dominated by $O(AP^{2})$. For instance, with parameters set to A=2, P=30, N=10, $\tilde{N}=30$, J=200, and I=100, the power calculation takes approximately 2.48 min on a local machine configured with a 14‐core socket cluster.

## 5 Some Considerations About Cross‐Validation

Since the PLS model is completely defined once the number of score components is specified and cross‐validation is usually applied to determine that number, cross‐validation plays a key role in PLS applications (Wold 1978). The PLS algorithm, which converges to the least squares solution when the maximum number of score components is used, is usually stopped early to balance bias and variance, thereby enhancing model generalizability (Stocchero et al. 2022). One of the most commonly used rules for optimizing PLS models is to select the number of score components (i.e., the number of iterations of the PLS algorithm) that yields the first maximum or minimum of the test statistics calculated in cross‐validation under the constraint to pass the permutation test. However, alternative methods have been proposed, such as using the full training data without subsampling to determine the optimal number of score components. For example, analyzing the significance of eigenvalues from the PLS eigenproblem seems promising (Wiklund et al. 2007; Stocchero 2023). Cross‐validation is also widely used to estimate predictive power in calibration problems and to calculate confidence intervals for PLS model parameters.

The same test statistics computed on the training data can also be estimated using cross‐validation. For classification problems, the MCC and score‐based test can be calculated by analyzing the classes and predictive scores for the out‐of‐bag samples used in cross‐validation, respectively. In contrast, considering the regression part of the model, $R^{2}$ can be calculated using cross‐validation results, obtaining the so‐called $Q^{2}$ statistic. As a general behavior, these test statistics show values smaller than or equal to those estimated on the whole training set.

The most common cross‐validation strategy in PLS is K‐fold cross‐validation, where K typically decreases as the number of observations increases. Groups are stratified to reflect the training data structure, and repeated or double cross‐validation is used to address data substructures when the data set size allows. It is worth noting that the values of test statistics depend on the type of cross‐validation implemented. Indeed, different numbers of groups may lead to different values of the test statistics, and their trends with respect to the number of PLS‐score components may be different, leading to different optimal numbers of score components. The test statistics for the whole training data and those from cross‐validation should align closely in the presence of well‐behaved PLS models. Significant discrepancies may indicate overfitting, leading to reduced generalizability.

When a randomization test is applied, the p‐values of the test statistics calculated by cross‐validation can be estimated following the same procedure described in Section 3 for the whole training data. In our experience with PLS, p‐values in cross‐validation are generally smaller than or equal to those from the training data when analyzing highly correlated, redundant, and noisy data sets with more features than observations. This behavior may occur because PLS overfits the training data when its structure is perturbed by permutation, resulting in poor predictions for out‐of‐bag data. Consequently, the power of test statistics in cross‐validation is often higher than that calculated from the whole training data.

Based on these considerations, we briefly discuss the power and sample size estimation using test statistics calculated by cross‐validation. The same procedure presented in Section 4 can be applied to MCC, score‐based test, and $R^{2}$ obtained via cross‐validation. Suppose model parameters estimated under cross‐validation align closely with those from the whole data set. In that case, the score structure from cross‐validation may be used for data simulation under the alternative hypothesis, eventually removing the orthogonality constraint between simulated scores. Since effects estimated via cross‐validation are typically smaller than or equal to those from the training data, power will generally be lower for a given model. However, for well‐behaved PLS models, these differences should be minimal. Significant discrepancies may lead to unreliable simulated data, in which case only scores from the whole training data should be used.

In addition, power and sample size depend on the type of cross‐validation, and with the increase in the sample size, the chosen cross‐validation technique may become suboptimal. However, since the p‐values from cross‐validation are generally smaller than those from the training data, sample size decisions should be driven by the test statistics of the whole training data. As a preliminary conclusion, if test statistics from cross‐validation and training data are similar, power and sample size estimations based on the entire data set should provide reasonable results. However, given the relevance of this topic in the PLS framework, further investigations that are out of the aim of the present study are requested, and a dedicated investigation will be performed in the future.

## 6 Applications to Data

Simulated data and two real data sets are investigated here. Calculations were performed using a scientific computing cluster with a processor having 20 CPU and 200 GB of RAM and the R package called powerPLS available on CRAN (https://CRAN.R‐project.org/package=powerPLS).

We use I=100 simulations under the alternative hypothesis and set α=0.05 for all analyses. In Section 6.2, J=500 permutations are employed, while in Section 6.1, we reduce this to J=200 to expedite computation time.

### 6.1 Simulated Data

The main advantage of considering simulated data is that their structure is a‐priori known. This study considers a 2‐class problem with a pilot data set composed of $N_{g}=5\;\;\forall g\in \left\{1,2\right\}$ observations per class and P=30 predictor variables. The simulated data set has been built, imposing the following data structure: five predictor variables closely related to the class membership and 25 noisy ones. Specifically, the matrix $X_{\text{pilot}}\in R^{10\times 30}$ of the pilot data has been simulated as the following block matrix:

(4) $$X_{\text{pilot}}=\left[T_{\text{pilot}}P_{\text{pilot}}^{⊤}|X_{\bar{R}}\right],$$

where $\left[T_{\text{pilot}}P_{\text{pilot}}^{⊤}\right]\in R^{10\times 5}$ is associated to the class and $X_{\bar{R}}\in R^{10\times 25}$ contains random noise. The matrix $T_{\text{pilot}}\in R^{10\times A_{\text{pilot}}}$ is the PCA‐score matrix of $C=[C_{1}^{⊤}|C_{2}^{⊤}]$ where $C_{1}∼N\left(0,I_{A_{\text{pilot}}}\right)$ and $C_{2}∼N\left(\mu ,I_{A_{\text{pilot}}}\right)$ with μ∈{2,5}, $P_{\text{pilot}}\in R^{5\times A_{\text{pilot}}}$ is the PCA‐loading matrix of a $\left(A_{\text{pilot}}\times 5\right)$ matrix sampled from a U(0,1), and $X_{\bar{R}}$ is sampled from a $N(0,I_{25})$. The parameter μ, which defines the distance between the centers of the distributions of the two classes, is used to set the effect. Indeed, large values of μ can be interpreted as large effects. The dimension $A_{\text{pilot}}$ has been set to 2 (the results for $A_{\text{pilot}}=3$ are reported in Appendix C).

**FIGURE 1:**
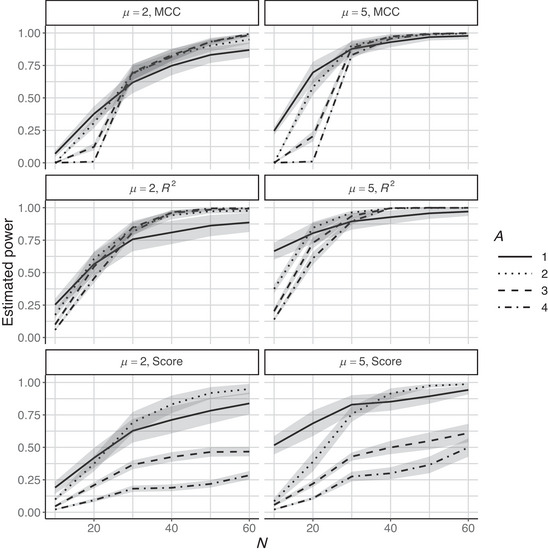
Estimated power across different sample size $\tilde{N}\in \left\{10,20,30,40,50,60\right\}$ using the test statistics T introduced in Section 3. The pilot data have been simulated with $A_{\text{pilot}}=2$, whereas the power has been estimated considering a PLSc model with A∈{1,2,3,4} score components. Different linetypes have been used to represent the power curves for different A. The shadow areas represent the corresponding confidence intervals at level 0.95. One hundred MC simulations and 200 permutations have been considered for each analysis.

Figure 1 shows the mean of the estimated power across 30 simulations of the pilot data following the procedure defined in Equation (4). For each of the 30 simulations, the power has been estimated following the procedure described in Algorithm 1. The number of observations per class of the new data sets simulated from the pilot data following the procedure described in Subsection 4.1 was ${\tilde{N}}_{1}={\tilde{N}}_{2}\in \left\{5,10,15,20,25,30\right\}$, and the number of score components as A∈{1,2,3,4}. The upper panel of Figure 1 refers to the results using MCC as test statistic, the center panel to the results obtained considering the squared Pearson correlation coefficient $R^{2}$ between the dependent variable mapped into the Euclidean space and the estimated one, and the bottom panel to the results when the test statistic based on $T_{p}$ is used in the power analysis estimation process.

Figure 1 shows that power increases with increasing sample size, as expected. Moreover, in the case of small sample sizes, the hypothesis test based on the MCC‐derived test statistic exhibits lower power compared to using the other two statistics analyzed. For example, fixing $\tilde{N}=10$ and considering the largest effect size (i.e., μ=5) and one score component, the mean across 30 simulations of the estimated power equals 0.168 if MCC is considered. In contrast, it equals 0.302 and 0.411 if the score‐based test and the $R^{2}$ are employed, respectively. This result confirms the intuition of Rosenblatt et al. (2021), which attributes the loss of power in the hypothesis test based on the MCC‐derived statistic to the discretization inherent in the MCC, compared to tests that exploit the continuous nature of the classification model under analysis. It is well‐known that using permutation theory to compute the null distribution of a discrete test statistic is conservative (Hemerik and Goeman 2018; Rosenblatt et al. 2021). However, the conservativeness generally decreases with sample sizes (Kim et al. 2016) as we can note in Figure 1. In fact, looking at the results corresponding to large N, the hypothesis test based on the MCC test statistic gains power with respect to the one based on the predictive scores, particularly if A∈{3,4}.

**FIGURE C1:**
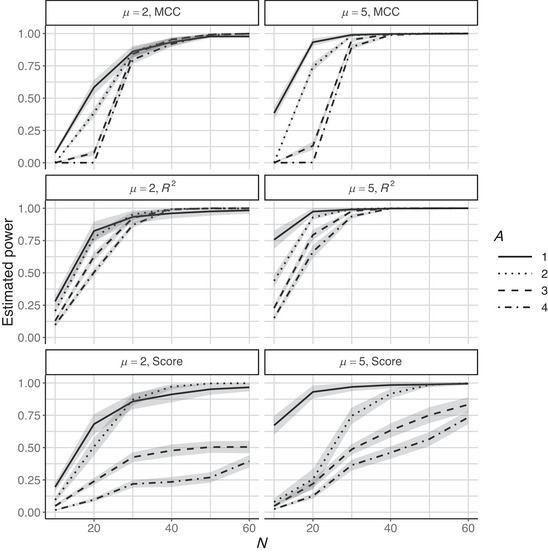
Estimated power across different sample size N∈{10,20,30,40,50,60} using the test statistics T introduced in Section 3. The pilot data have been simulated with $A_{\text{pilot}}=3$, whereas the power has been estimated considering a PLSc model with A∈{1,2,3,4} score components. Different linetypes have been used to represent the power curves for different A. The shadow areas represent the corresponding confidence intervals at level 0.95. One hundred MC simulations and 200 permutations have been considered for each analysis.

**FIGURE C2:**
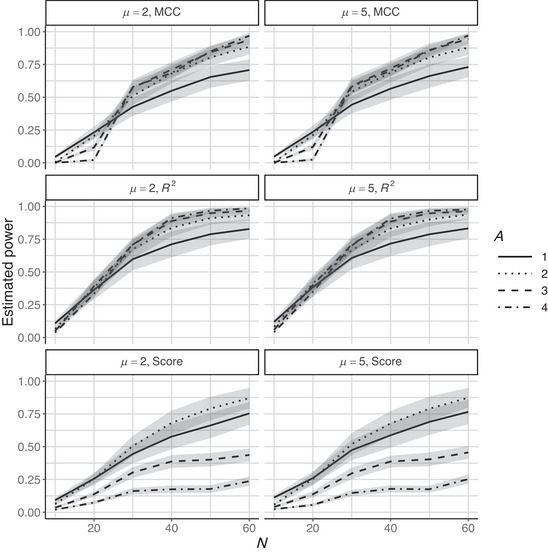
Estimated power across different sample size N∈{10,20,30,40,50,60} using the test statistics T introduced in Section 3. The pilot data have been simulated with $A_{\text{pilot}}=3$, whereas the power has been estimated considering a PLSc model with A∈{1,2,3,4} score components. Different linetypes have been used to represent the power curves for different A. The shadow areas represent the corresponding confidence intervals at level 0.95. One hundred MC simulations and 200 permutations have been considered for each analysis.

The behavior of the power curve is not only dependent on the test statistic but also heavily dependent on the number of score components of the PLSc model. For MCC and $R^{2}$, the curves are similar in the region of large sample size, independently of the number of score components, but show differences in the area of small sample size. Indeed, for small sample sizes, PLSc may overfit the data modeling noise with the effect of decreasing power. This effect decreases with the increase in the sample size. For the score‐based statistic, the curves are different, and the behavior is more complex. The same trends are observed in Figure C1 obtained considering pilot data with $A_{\text{pilot}}=3$ and in Figure C2 in the case of skewed predictor variables.

Regardless of the choice of test statistic, the sample size estimation heavily depends on the number of score components, which must be accurately determined to obtain a reliable estimate.

### 6.2 Aqueous Humor Data

The data set has been extracted from the data published by Locci et al. (2019) and is available in the R package powerPLS (https://CRAN.R‐project.org/package=powerPLS), where 59 postmortem aqueous humor samples were collected from closed and opened sheep eyes. Each sample was analyzed by 1H NMR spectroscopy, obtaining the quantification of 43 metabolites. As a result, a pilot data set composed of 59 observations (29 from opened eyes and 30 from closed ones) and 43 predictor variables was obtained. More details about sample collection, experimental procedure, and data preprocessing can be found in Locci et al. (2019). Data were autoscaled before performing data analysis. In power analysis, the residuals of the PLSc model were submitted to PCA to obtain a score structure able to explain at least the 80% of the total variance of the pilot data.

The two groups of samples, corresponding to opened and closed eyes, were investigated by PLSc. Considering A∈{1,2,3,4} score components, the models with the test statistics reported in Table 1 were obtained. The adjusted p‐values were less than 0.05 for all the statistics. As expected, all the test statistics increased with the increase in the number of components.

**TABLE 1:** Aqueous Humor data: Estimated test statistics (as defined in Section 3) for the pilot data considering A∈{1,2,3,4} number of score components.

A	**MCC**	**Score**	$R^{2}$
1	0.83	10.4	0.66
2	0.87	12.7	0.74
3	0.93	14.8	0.79
4	0.97	15.7	0.81

The power was greater than 0.90 for each model, independently of the test statistic used and the number of score components A considered. During the data simulation, the level of similarity between the pilot data and the data simulated under the alternative hypothesis was assessed by computing two measures of association between matrices, that is, the RV coefficient (Escoufier 1973) and the Procrustes one (Gower 1971). Both indices take values in [0,1], where 0 stands for the absence of association (i.e., orthogonal information) while 1 equals complete similarity between the two data matrices. Considering the whole set of simulated data, the RV index was at least equal to 0.83, while the Procrustes one showed a minimum equal to 0.94. These results proved that the covariance structure was preserved during power estimation.

Calculating the power curves for 8,12,20,30,42 observations per class, we found that the power curves obtained considering the $R^{2}$ and score‐based test statistics showed greater power than MCC. Specifically, considering, for instance, a PLSc model with two score components, a sample size of approximately 16 observations per class is required for the hypothesis test using MCC to have a power of 0.80, whereas a sample size of approximately 10 observations per class is requested both for the hypothesis tests using $R^{2}$ and score‐based test statistics.

### 6.3 Wheezing Data

The data set has been generated starting from the data investigated in Carraro et al. (2018) and available in the R package powerPLS (https://CRAN.R‐project.org/package=powerPLS). The study aimed to discover differences in the urinary metabolome capable of distinguishing children developing early‐onset asthma and children with transient wheezing. Specifically, urine samples from 16 subjects for each group were collected and analyzed using untargeted metabolomics based on mass spectrometry. In the present study, the raw data acquired in positive ionization mode were extracted, and the features obtained were submitted to the procedure introduced in Stocchero (2020) to discover the relevant features. The feature selection procedure was applied to avoid the irrelevant features covering the effects of the relevant ones. As a result, a pilot data set composed of 174 features (9% of the total features extracted) and 32 observations, 16 in each group, was obtained. More details about the experimental design, sample collection and preparation, and metabolomics investigation can be found in Carraro et al. (2018). Data were autoscaled before performing data analysis.

PLSc compared the two groups of children. As in Subsection 6.2, Table 2 shows the values of the three test statistics proposed in Section 3. All the hypothesis tests were significant under A=1, while if A=2, only the hypothesis tests using the $R^{2}$ and score‐based test statistics were significant.

**TABLE 2:** Wheezing data: estimated test statistics (as defined in Section 3) for the pilot data considering A∈{1,2,3,4} number of score components.

A	**MCC**	**Score**	$R^{2}$
1	0.88	6.9	0.61
2	1	13.1	0.85
3	1	19.3	0.93
4	1	36.1	0.98

In power calculation, the residuals of the PLSc model were submitted to PCA to obtain a score structure explaining at least the 80% of the total variance of the pilot data. The power estimated considering one score component, and MCC was 0.76, whereas it was 0.95 both using score‐based and $R^{2}$ test statistics. Across the data simulations, the level of similarity calculated by the RV (Escoufier 1973) and Procrustes (Gower 1971) indices was greater than 0.89 and 0.94 respectively.

The power curves were calculated considering 8, 12, 16, and 24 observations per group. In the case of MCC, the power was greater than 0.80 when the number of observations per group was greater than 19. In contrast, at least 14 observations per group were necessary to have a power greater than 0.80 using the score‐based and $R^{2}$ test statistics.

## 7 Concluding Remarks

We have introduced an innovative procedure for conducting power analysis within the context of PLS‐based methods.

The proposed approach leverages the score structure identified in the pilot data when simulating data under the alternative hypothesis to estimate power across varying sample sizes. It considers explicitly the data decomposition discovered by PLS and can be applied in principle both to regression and to classification problems. Following the strategy introduced in Section 4.1, the correlation structure of the pilot data has been preserved during data simulation, as proved by investigating the real data sets in Section 6.

For the sake of simplicity, 2‐class classification problems were investigated, testing the null hypothesis of no differences between classes. Specifically, we have introduced three permutation‐based test statistics to analyze the covariate distribution between the two classes. The approach uses test statistics that can also be estimated in the case of data with a small sample size (i.e., when less than 10–15 observations per class are available), even if for larger pilot data, cross‐validation may be used to estimate the test statistics to use in the statistical test procedure. The use of test statistics calculated by cross‐validation has been briefly discussed, even if further studies are requested to investigate this scenario better.

To evaluate the effectiveness of our proposed power analysis approach, we conducted simulations across various scenarios and analyzed two real data sets.

In all cases, the power curve increased with the increase in the sample size, as expected. Interestingly, the power curve seems to depend heavily on the number of score components used in PLS. Consequently, estimating the correct number of scores to use in PLS modeling is fundamental to obtaining reliable power and sample size estimation. In principle, if test statistics based on the estimated matrix of the regression coefficients are used, that is, MCC and $R^{2}$, the greater the number of score components is, the greater the differences detected between classes are, that is, MCC and $R^{2}$ increase, but then power may not increase. Indeed, overfitting may be present when an excessive number of score components is used, increasing the p‐values estimated under the null hypothesis and decreasing the power. Moreover, the correction for FWER may limit the effect of increasing the number of scores, reducing the significance level of the test.

The $R^{2}$ test statistic seems to be a better candidate than MCC since it increases the power of the hypothesis test, at least in the case of small pilot data sets. However, $R^{2}$ may be misleading in the case of classification problems because small and large residuals in the calculation of the dependent variable may be associated with the same class, making $R^{2}$ an unreliable parameter to measure the goodness in classification, that is, small $R^{2}$ may be associated to large MCC. Some type of regularization, for example, following the $dQ^{2}$’s idea (Westerhuis et al. 2008), could be necessary to adapt $R^{2}$ to classification, which will be a further research direction. Moreover, both MCC and $R^{2}$ can be used to study more general multiclass problems. Still, a new score‐based test statistic must be introduced for a general G‐class problem since G−1 predictive scores are calculated.

The present study must be considered a preliminary study since it does not address all the issues of power analysis, even if it draws a methodology toward a comprehensive approach. Moreover, it is worth noting that the same lines of thought presented for the 2‐class classification problem can be adapted to deal with more complex problems, that is, multiclass and regression scenarios. Section 2.1 outlines the PLSR, while the PLSc model defined in Section 2.2 follows the generalized approach of Stocchero et al. (2021). For simplicity, the $R^{2}$ test statistic can be used for both classification and regression problems, and the process for simulating data under the alternative hypothesis remains the same as explained in the final part of Subsection 4.1. Finally, power and sample size will be estimated again following Algorithms 1 and 2.

The main limit of the study is that the effect size has not been considered as a parameter to be investigated in power analysis. A possibility could be to use a metric based on Hotelling's t‐squared statistic to define the effect size. Indeed, since the new data were simulated to preserve the correlation structure of the pilot data, the effect size was maintained unchanged during the power calculation. It is not trivial how to define and measure the effect size in PLS‐based methods; a dedicated study will deal with this topic. However, the data decomposition in predictive and nonpredictive parts generated by PLS also paves the way for the possibility of defining and modifying the effect size for a more general power analysis. Indeed, a natural approach may be changing the predictive score structure to increase or decrease the effect size, leaving the nonpredictive part unchanged, but this will be discussed in a further study.

Another limitation is that the power in the estimation of the number of PLS‐score components and that of the relevant features discovered by PLS were not considered here. Even if these two points will be discussed in the future, we want to disclose that the methodology proposed here can also be adapted to address these issues.

## Author Contributions

**Angela Andreella:** Conceptualization, methodology, software, formal analysis, investigation, writing–original draft. **Livio Finos:** conceptualization, methodology, supervision, writing–review & editing. **Bruno Scarpa:** conceptualization, supervision. **Matteo Stocchero:** Conceptualization, methodology, software, formal analysis, investigation, writing–original draft.

## Conflicts of Interest

The authors declare no conflicts of interest.

### Open Research Badges

This article has earned an Open Data badge for making publicly available the digitally‐shareable data necessary to reproduce the reported results. The data is available in the Supporting Information section.

This article has earned an open data badge “**Reproducible Research**” for making publicly available the code necessary to reproduce the reported results. The results reported in this article were reproduced partially due to computational complexity.

## Supporting information

Supporting Information

## Data Availability

Data are available in the R package called powerPLS available on CRAN (https://CRAN.R‐project.org/package=powerPLS).

## Appendix A Partial Least Squares Algorithm

Several algorithms have been proposed for PLS regression. Here, we report the so‐called “eigenvalue” PLS2 algorithm fixing the number of latent components equals A.

ALGORITHM A1 “eigenvalue” PLS2 algorithm **Table:**

**Require:** $X\in R^{N\times P}$; $Y\in R^{N\times K}$; A	
1:	${\hat{E}}_{0}=X$
2:	${\hat{F}}_{0}=Y$
3:	**for** a in 1,⋯,A **do**
4:	${\hat{E}}_{a-1}^{⊤}{\hat{F}}_{a-1}{\hat{F}}_{a-1}^{⊤}{\hat{E}}_{a-1}w_{a}=\lambda_{a}w_{a}$ ▹ Estimate $w_{a}$
5:	$t_{a}={\hat{E}}_{a-1}w_{a}$
6:	$Q_{t_{a}}=I_{N}-t_{a}{\left(t_{a}^{⊤}t_{a}\right)}^{-1}t_{a}^{⊤}$
7:	${\hat{E}}_{a}=Q_{t_{a}}{\hat{E}}_{a-1}$ ▹ X‐deflation step
8:	${\hat{F}}_{a}=Q_{t_{a}}{\hat{F}}_{a-1}$ ▹ Y‐deflation step
9:	**end for**

The matrices ${\hat{E}}_{a}$ and ${\hat{F}}_{a}$ are called the residual matrix of the X‐ and Y‐block, respectively, the vectors $w_{a}$ and $t_{a}$ are called weight vector and score vector, respectively, and $Q_{t_{a}}$ is an orthogonal projection matrix that projects a given vector into the space orthogonal to $t_{a}$. We denote $\hat{E}:={\hat{E}}_{A}$ and $\hat{F}:={\hat{F}}_{A}$. When the calculation of the weight vector in step 4 of Algorithm A1 is replaced by a given vector $w_{a}$ defined as input of the algorithm, the algorithm becomes the “Iterative Deflation Algorithm” (IDA), which is a general algorithm able to solve the least squares problem ${\hat{B}}_{\text{LS}}=\arg\min_{B}||Y-{XB||}_{F}^{2}$ for a nontrivial choice of the vectors $w_{a}$ (Stocchero 2019). The main properties of the “eigenvalue” PLS2 algorithm have been extensively discussed in the past literature. Readers can refer to Höskuldsson (1988) and to Stocchero (2019).

## Appendix B Posttransformation of PLS2

Post‐transformation of PLS2 has been introduced in Stocchero and Paris (2016) to separate the structured data variation discovered by PLS2 into the predictive and nonpredictive parts. From a geometrical point of view, post‐transformation linearly transforms the score space of the PLS2 model spanned by T to obtain two new sets of scores: the predictive scores $T_{P}$ able to explain the dependent matrix Y, and the nonpredictive scores $T_{o}$ that are orthogonal to Y, that is, $\left[T_{P}T_{o}\right]=T\tilde{G}$ where the score matrices $T_{P}=\left[t_{P}\right]$ and $T_{o}=\left[t_{o}\right]$ have been introduced. The matrix $\tilde{G}$ is a suitable nonsingular matrix.

Post‐transformation is performed using the columns of WG, where G is a suitable orthogonal matrix, as weight vectors within the IDA instead of the weight vectors $w_{a}$ calculated by PLS2. The algorithm to calculate the matrix G required to posttransform the PLS2 model is reported in Algorithm B1.

ALGORITHM B1 Algorithm to calculate the matrix G
**Table:**

**Require:** $X\in R^{N\times P}$; $Y\in R^{N\times K}$; $W\in R^{P\times A}$	
**Ensure:** $G\in R^{A\times A}$	
1:	$Y^{⊤}XW=USV^{⊤}$ ▹ Singular Value Decomposition
2:	$\left(I_{A}-VV^{⊤}\right)g_{o_{i}}=\lambda_{o_{i}}g_{o_{i}}$ ▹ M positive eigenvalues
3:	$G_{o}=\left[g_{o_{1}}\cdots g_{o_{M}}\right]$
4:	$\left(I_{A}-G_{o}G_{o}^{⊤}\right)g_{P_{i}}=\lambda_{P_{i}}g_{P_{i}}$ ▹ A−M positive eigenvalues
5:	$G=[G_{o}g_{P_{1}}\cdots g_{P_{A-M}}]$

Alternative algorithms for calculating G have been proposed (Stocchero and Paris 2016). Moreover, since all the models based on IDA can be posttransformed (Stocchero 2019), procedures of posttransformation can be, in principle, developed for most of the PLS methods because most of the PLS‐based techniques are based on the IDA.

## Appendix C Simulated Data

Figure C1 has been obtained considering the same simulation process presented in Section 6.1 but setting the dimension $A_{\text{pilot}}=3$. The observations made regarding Figure 1 in Section 6.1 are equally relevant here. In particular, when dealing with small sample sizes, the hypothesis test based on the Matthews correlation coefficient (MCC) test demonstrates lower power compared to the ones based on the $R^{2}$ and scores test statistics.

Figure C2 was generated using the same simulation process described in Section 6.1, but with C simulated such that $C_{1}∼\text{lognormal}\left(0,I_{A_{\text{pilot}}}\right)$ and $C_{2}∼\text{lognormal}\left(\mu ,I_{A_{\text{pilot}}}\right)$. Similarly to Figure C1, the observations from Figure 1 are also relevant here.
