# Supplementary figures and images for: Toward Power Analysis for Partial Least Squares‐Based Methods
Source: Biom J. 2025 Mar 13;67(2):e70050. doi: 10.1002/bimj.70050 (PMC11905696; doi:10.1002/bimj.70050)

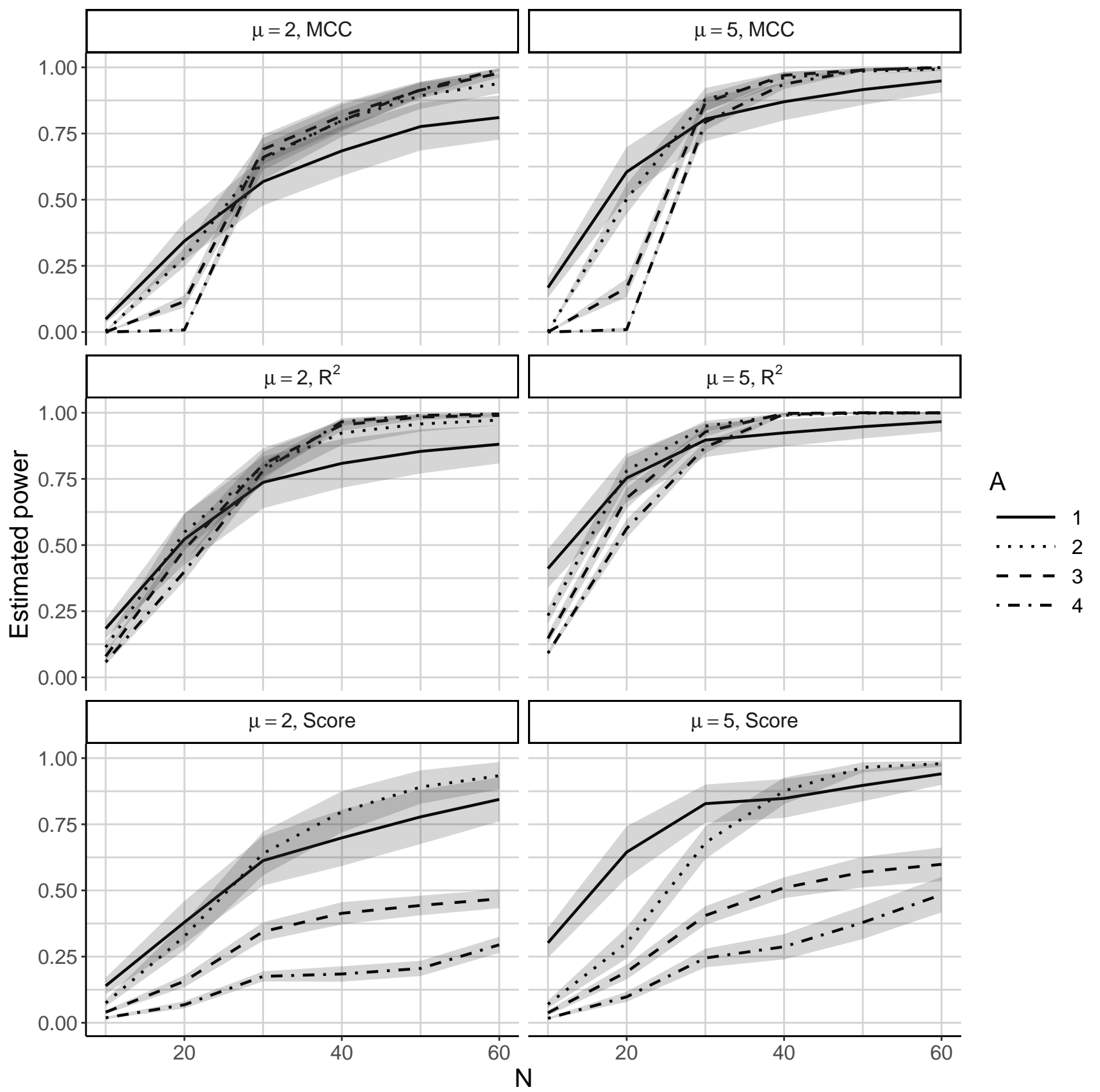

Supplement: Supplementary file 1 — Supporting Information [file BIMJ-67-e70050-s001.zip › Code_and_data/simulation/results/figure1.pdf]

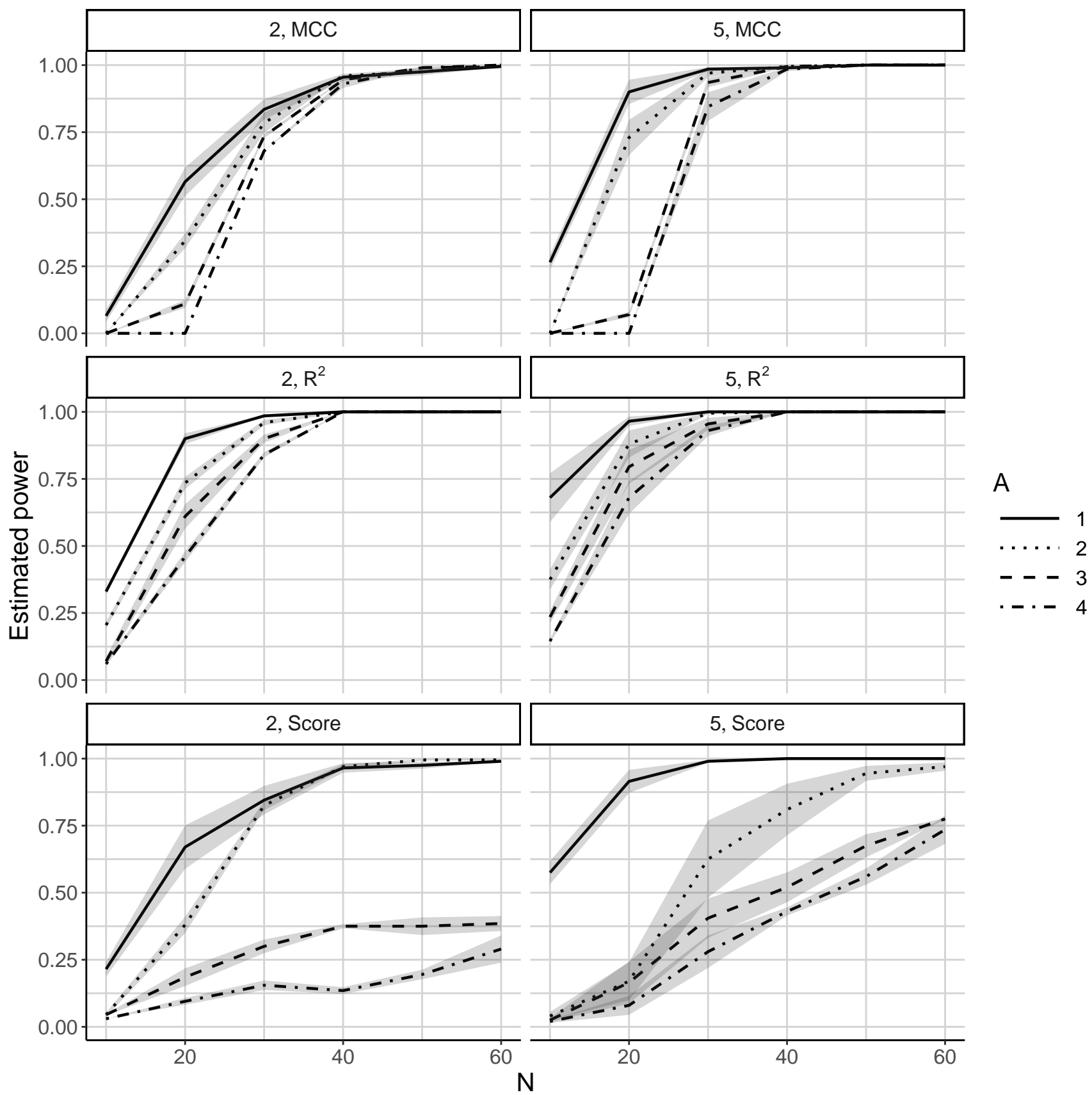

Supplement: Supplementary file 1 — Supporting Information [file BIMJ-67-e70050-s001.zip › Code_and_data/simulation/results/figure2.pdf]

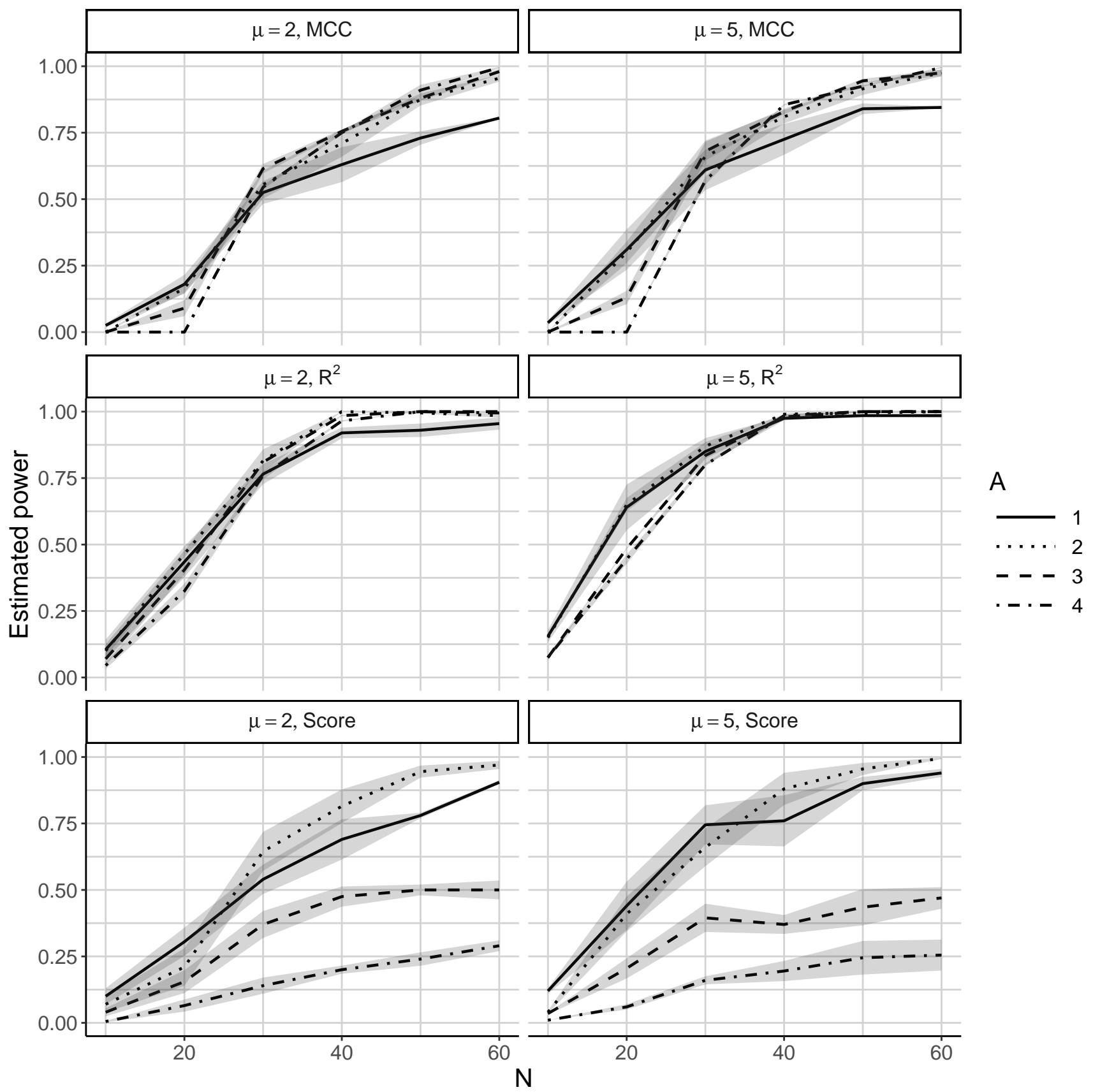

Supplement: Supplementary file 1 — Supporting Information [file BIMJ-67-e70050-s001.zip › Code_and_data/simulation/results/figure3.pdf]
